# A Survey of Imprinted Gene Expression in Mouse Trophoblast Stem Cells

**DOI:** 10.1534/g3.114.016238

**Published:** 2015-02-23

**Authors:** J. Mauro Calabrese, Joshua Starmer, Megan D. Schertzer, Della Yee, Terry Magnuson

**Affiliations:** * Department of Pharmacology, University of North Carolina, Chapel Hill, North Carolina 27599; † Lineberger Comprehensive Cancer Center, University of North Carolina, Chapel Hill, North Carolina 27599; ‡ Department of Genetics, University of North Carolina, Chapel Hill, North Carolina 27599; § Curriculum in Genetics and Molecular Biology, University of North Carolina, Chapel Hill, North Carolina 27599

**Keywords:** epigenetics, imprinting, microRNAs, noncoding RNA, trophoblast

## Abstract

Several hundred mammalian genes are expressed preferentially from one parental allele as the result of a process called genomic imprinting. Genomic imprinting is prevalent in extra-embryonic tissue, where it plays an essential role during development. Here, we profiled imprinted gene expression via RNA-Seq in a panel of six mouse trophoblast stem lines, which are *ex vivo* derivatives of a progenitor population that gives rise to the placental tissue of the mouse. We found evidence of imprinted expression for 48 genes, 31 of which had been described previously as imprinted and 17 of which we suggest as candidate imprinted genes. An equal number of maternally and paternally biased genes were detected. On average, candidate imprinted genes were more lowly expressed and had weaker parent-of-origin biases than known imprinted genes. Several known and candidate imprinted genes showed variability in parent-of-origin expression bias between the six trophoblast stem cell lines. Sixteen of the 48 known and candidate imprinted genes were previously or newly annotated noncoding RNAs and six encoded for a total of 60 annotated microRNAs. Pyrosequencing across our panel of trophoblast stem cell lines returned levels of imprinted expression that were concordant with RNA-Seq measurements for all eight genes examined. Our results solidify trophoblast stem cells as a cell culture-based experimental model to study genomic imprinting, and provide a quantitative foundation upon which to delineate mechanisms by which the process is maintained in the mouse.

Genomic imprinting is an epigenetic process initiated during mammalian gametogenesis, which results in preferential expression of genes from one parentally inherited allele over the other. More than one hundred fifty imprinted genes have been identified in mammals (Williamson *et al.* 2013). As a class, these genes play important roles in development, growth, metabolism, and social adaptation (Ferguson-Smith 2011; Garfield *et al.* 2011). Defects in imprinting can cause cancer, in the form of Wilm’s tumor, and other human diseases, including Angelman, Prader-Willi, Beckwith-Wiedemann, and Silver-Russell syndromes (Butler 2009). Faithful maintenance of imprinting also plays an important role in reprogramming and maintenance of stem cell identity (Zacharek *et al.* 2011; Stadtfeld *et al.* 2012).

Genomic imprinting is critical and prevalent in the placenta, consistent with its essential role in development. Parthenogenetic embryos with no contribution from the paternal genome die early in gestation with severe defects in the trophectoderm, the pool of cells that give rise to the placenta (Surani and Barton 1983). Genomic imprinting is required in placental tissue as early as, or before, embryonic gestational day 6.5 (Clarke *et al.* 1988). Moreover, much of the tissue-specific imprinting that is known to occur is found in the placenta (Wang *et al.* 2011b; Prickett and Oakey 2012; Wang *et al.* 2013b; Court *et al.* 2014), again underscoring its importance for extra-embryonic development.

The study of genomic imprinting has helped to define paradigms of epigenetic regulation and long noncoding RNA (lncRNA) function in mammals (Barlow 2011; Ferguson-Smith 2011). For example, parent-of-origin−specific DNA methylation deposited at imprinted control regions during gametogenesis is a master regulator of imprinted states. Accordingly, genomic imprinting has served as an important model to understand the deposition, propagation, and biological function of DNA methylation in development and organismal homeostasis (Kelsey and Feil 2013). In addition to DNA methylation, several imprinted genes also require lncRNAs to propagate their allelic epigenetic states, or, are themselves lncRNAs (Lee and Bartolomei 2013). Indeed, some of the earliest lncRNAs identified, *H19* and *Kcnqot1*, are imprinted and were discovered because of their strong associations with human disease (Lee and Bartolomei 2013). The study of imprinted lncRNAs will continue to provide important paradigms as newly described lncRNAs emerge as essential regulators of diverse physiological processes.

Considering the importance of genomic imprinting in health and disease, its prevalence in placental tissue, and its paradigmatic role in defining mechanisms of epigenetic regulation and lncRNA function, it remains a high priority to develop appropriate experimental models to study the process in extra-embryonic tissues. Mouse trophoblast stem cells (TSCs) offer one such experimental framework. TSCs are *ex vivo* derivatives of the trophectodermal stem cell population that mediates implantation and gives rise to the placenta, and they provide a renewable, extra-embryonic−derived cell population free of maternal tissue contamination (Quinn *et al.* 2006). Furthermore, they are easily propagated in culture. As a result, TSCs are amenable to large-scale genomic and biochemical studies, and their transcriptional outputs can be modified via overexpression, knockdown, or precision genome-editing approaches.

Here, we profiled allele-specific gene expression via RNA-Seq in a panel of six F1-hybrid mouse TSC lines. We detected parent-of-origin (PO) biased expression of 48 genes, an equal number of which were expressed with maternal and paternal biases, respectively. Thirty-one of these were known imprinted genes, whereas 17 had not been previously reported to exhibit PO expression bias and could be considered candidate imprinted genes. Sixteen of the 48 PO-biased genes were known or putative lncRNAs. Further, six of the known imprinted genes expressed in TSCs encode for a total of 60 known microRNAs. PO biases in gene expression detected via RNA-Seq were concordant with those detected via pyrosequencing for eight genes examined across six profiled TSC lines. Our results provide a quantitative foundation upon which to dissect mechanisms that underpin PO biased gene expression in mouse TSCs.

## Materials and Methods

### TSC derivation and culture

TSCs were derived and propagated in an undifferentiated state using protocols described in (Quinn *et al.* 2006).

### RNA-Seq

Before RNA extraction with Trizol, TSC lines were passaged twice off of irradiated feeder cells. For these two passages, TSCs were cultured in 70% feeder-conditioned media plus growth factors, as described in (Quinn *et al.* 2006). At each passage after trypsinization, TSC suspensions were preplated for 30 min to deplete irradiated feeder populations. CB.1 and BC.1 RNA-Seq data were collected in Calabrese *et al.* (2012). cDNA libraries for CB.2, CB.3, BC.2, and BC.3 TSC lines were prepared in this work, from 4 µg of total TSC RNA using Kapa Biosystem’s Stranded mRNA-Seq kit, which maintains strand information and enriches for poly-adenylated transcripts via oligo dT bead purification. cDNA libraries prepared from CB.2, CB.3, BC.2, and BC.3 TSC lines were sequenced once on Illumina’s HiSeq and once on Illumina’s NextSeq500 instruments, respectively, and data were pooled per cell line to derive final allele-specific expression ratios. Total read counts obtained per TSC line were as follows: [CB.1, 69,788,067]; [CB.2, 123,636,335]; [CB.3, 135,130,738]; [BC.1, 60,678,597]; [BC.2, 123,714,166]; [BC.3, 150,370,343]. Sequence data collected as part of the present study were deposited in Gene Expression Omnibus (GEO) database under accession number GSE63968.

### Allele-specific read counts

Allele-specific read counts per gene were determined as in Calabrese *et al.* (2012). In brief, Cast single-nucleotide polymorphisms (SNPs) from Keane *et al.* (2011) were substituted into their corresponding mm9 genomic positions to create an *in silico* Cast genome, and SNP-overlapping reads that uniquely aligned to either the B6 (mm9) or Cast genomes were retained (Kent *et al.* 2002). A nonredundant list of mouse genes was annotated from the set of UCSC Known Genes as in Calabrese *et al.* (2012) via the use of the longest exemplar per gene to count allele-specific expression. In addition to The University of California Santa Cruz (UCSC) Known Genes, uniquely aligning RNA-Seq reads from CB.1 and BC.1 TSCs that did not match in strand with, or were not located within ±5 kb of, any UCSC Known Gene, were selected for clustering to approximate newly annotated transcriptional units. Units reported represent strand-matched reads falling within ±5 kb of each other. Allele-specific counts represent the total number of SNP-overlapping reads that uniquely mapped between the start and end of each gene or transcriptional unit, including intronic regions.

### Calculation of significance of PO bias

Allelic read counts for all autosomal genes were imported into edgeR and normalized using edgeR’s counts per million (CPM) metric. Only genes whose normalized allele-specific counts summed to more than 1 CPM in each of the six profiled TSC lines were tested for differential allelic expression. Differential expression between Cast and B6 alleles was tested separately in the CB.x and BC.x TSC lines, such that each group of F1-hybrid TSC lines was represented by three biological replicates: CB.1, 0.2, and 0.3 for the CB.x lines, and BC.1, 0.2, and 0.3 for the BC.x lines. Differential expression between Cast and B6 alleles within each F1-hybrid group was tested via edgeR’s generalized linear model likelihood ratio test, and *P*-values from both tests were adjusted to false discovery rates using the Benjamini-Hochberg method (Robinson *et al.* 2010). Genes exhibiting PO biases with false discovery rates scores of ≤ 0.05 in both CB.x and BC.x cell lines were considered to be significantly biased.

### Calculation of total gene expression levels

Total (*i.e.*, allele-nonspecific) gene expression levels were approximated for all UCSC Known Genes in each TSC line using the Tophat and Cufflinks algorithms and are reported using the reads per kilobase per million aligned reads (RPKM) metric; for newly annotated transcription units (*e.g.*, the *Tsci* transcripts), exonic coordinates were not clearly apparent, and thus reads that matched in strand and fell between the start and end of the unit were used to calculate RPKM via custom scripts.

### Pyrosequencing

PCR primers for individual pyrosequencing assays were designed to amplify a less than 200 base pair exonic region surrounding a known SNP for each of eight genes (Table 1). Sequencing primers were either directly adjacent to the SNP or one base pair removed. To perform pyrosequencing assays, 5 µg of RNA from each TSC line was reverse transcribed with Superscript III (Invitrogen). PCR amplification from cDNA was performed with Apex Taq DNA Polymerase (Genesee Scientific) and the cycle number shown in Table 1 using the following PCR conditions: 95° for 30 sec, 56° for 30 sec, and 72° for 30 sec. The PyroMark Q96 MD Pyrosequencer (Biotage, AB), PyroMark Gold Q96 CDT Reagents (QIAGEN), and Streptavidin Sepharose beads (GE Healthcare) were used for pyrosequencing. Quantification of allele-specific expression was performed using PyroMark Q96 MD software. Box and whisker diagrams were generated using matplotlib version 1.4.2.

**Table 1:** Primers used for pyrosequencing

Gene	Forward Primer	Reverse Primer	Sequencing Primer	B6	Cast	SNP Position	PCR
*Igf2r*	CCAGAGGACACACAGCTGAA*^a^*	CACTGTGACCCTCTGGTGAA	CCCAGAGTTCAGCCACGAGA	G	A	chr17: 12879541	28×
*Igf2*	GGGTGTCAATTGGGTTGTTT	AGGGACAGTTCCATCACGTC*^a^*	AATCAAATTTGGTTTTTTAGAA	C	T	chr7: 149839519	28×
*H19*	CCGAGACGATGTCTCCTTTG	GGTATAGCTGGCAGCAGTGG*^a^*	GAGGCCAGCCGCTTCTTCT	C	T	chr7: 149763597	28×
*AK017220*	CCTGATGACTGCAAACATGG*^a^*	CCAGCTTAGCCAACCTGAAG	GGTGGCTTCTGAAATCCTG	C	G	chr11: 51003537	32×
*Tsci6*	TCAGGAAAGTGAAGCGAGGT*^a^*	AAGGCAGTGATGGCAGAGAT	GGCCATGCATATCTCTTGC	A	G	chr15: 96990521	34×
*Gab1*	GGGGTCATTGGCACATAGTT	GAGGTGTCTCGGGTGAAGAG*^a^*	AGCTGCCGGAATGTTGTCG	A	C	chr8: 83308743	28×
*Qk*	CTCTGCCATGTCCTGGACTC	ACTAAAAACAAACCCACTTCCA*^a^*	CAGTACACACAGGTAATGT	T	C	chr17: 10404189	30×
*Dact2*	ACACCCTTCTGAATCGTTGC*^a^*	CAGAGTAGGCCCACACTTGC	CCACCATTGCCGAGACCAG	G	A	chr17: 14332970	34×

Single nucleotide polymorphism (SNP) positions are relative to mm9 genome build. Polymerase chain reaction (PCR), the number of PCR cycles used to amplify target genes in each assay.

a Location of biotin moiety.

## Results and Discussion

### Detection and validation of autosomal PO expression bias in TSCs

In a previous study, we generated a panel of F1-hybrid mouse TSC lines that were used to measure molecular properties of the inactive X-chromosome (Calabrese *et al.* 2012). These TSCs were derived from reciprocal crosses between two diverse, inbred mouse strains, CAST/EiJ (Cast) and C57BL/6J (B6). Using a high confidence, validated set of ~18 million informative SNPs from (Keane *et al.* 2011), allele-specific gene expression can be measured accurately in these cells by the counting of SNPs contained within uniquely mapping high-throughput sequencing reads (Calabrese *et al.* 2012).

**Figure 1:**
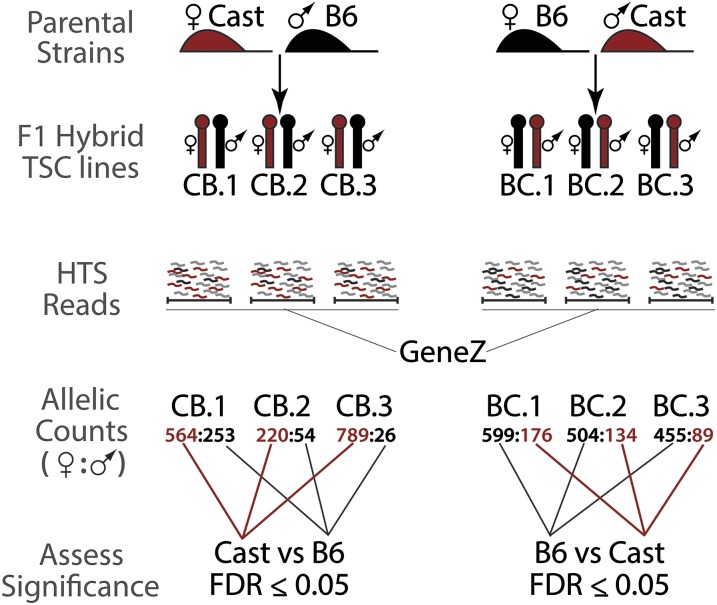
Strategy to profile PO expression bias in mouse TSCs. Strand-specific RNA-Seq was performed in six reciprocally derived F1-hybrid TSC lines. Informative SNPs contained within HTS reads that uniquely mapped to individual transcribed regions in each TSC line were summed and used to infer allele-specific expression bias. TSC lines of identical parentage were treated as biological replicates, and significant allelic expression biases were determined using EdgeR, correcting for multiple testing using the Benjamini Hochberg method. Allelic biases harboring an FDR ≤ 0.05 after multiple testing correction were deemed significant. PO, parent-of-origin; TSC, trophoblast stem cell; SNP, single-nucleotide polymorphism; HTS, high-throughput sequencing; FDR, false discovery rate.

To measure allelic biases in TSC gene expression, we analyzed strand-specific RNA-Seq data collected from six of these reciprocally derived F1-hybrid TSC lines: three lines were derived from a Cast mother and B6 father (referred to as “CB.x” lines) and three lines from a B6 mother and Cast father (referred to as “BC.x” lines). F1-hybrid TSC lines were grouped on the basis of their respective parentage, and significant allelic biases in gene expression in each group were detected using edgeR and corrected for multiple testing via the Benjamini-Hochberg method (Robinson *et al.* 2010) (Figure 1). To increase robustness of our significance calls, genes were only considered for allelic analysis if the sum of their allele-specific read counts was >1 CPM in all six profiled TSC lines. Under this CPM cutoff, 13,593 genes were eligible for allelic expression analysis (Supporting Information, Table S1).

In total, we detected 48 genes expressed with significant PO bias in TSCs. Twenty-four genes were expressed with a maternal bias and 24 with a paternal bias. This equal representation of PO bias between maternal and paternal genomes differed from that recently observed in mule and hinny placenta, where paternally biased genes predominated (Wang *et al.* 2013b). Thirty-one of the genes expressed with PO bias in TSCs were annotated previously as imprinted in the mouse (Table 2). To our knowledge, PO expression bias for the remaining 17 genes has not been previously described (Table 3).

**Table 2:** Known imprinted genes expressed with significant PO bias in TSCs

Gene	Chr	CB.1	CB.2	CB.3	BC.1	BC.2	BC.3	CBp	BCp	B	SNPs	RPKM
*AK076687**^a^*	chr2	5.3 (10, 180)	4.5 (26, 557)	0.4 (2, 551)	1.1 (2, 185)	1.8 (5, 269)	0.7 (3, 439)	5E-37	6E-62	P	226	1.03
*Sfmbt2*	chr2	2.3 (144, 6233)	8.4 (790, 8599)	1.6 (290, 17804)	1.0 (79, 7694)	1.5 (299, 19439)	1.0 (217, 22508)	8E-31	2E-50	P	1092	78.8
*Nespas**^a^*	chr2	0.0 (0, 94)	0.0 (0, 251)	2.2 (5, 223)	0.0 (0, 88)	4.0 (8, 191)	0.6 (1, 161)	4E-51	2E-30	P	34	2.58
*Phf17*	chr3	32.4 (665, 1387)	30.3 (2099, 4819)	46.8 (1568, 1786)	13.8 (130, 809)	24.7 (1483, 4515)	15.5 (820, 4457)	2E-03	2E-18	P	321	48.2
*Sgce*	chr6	4.5 (11, 231)	8.8 (78, 809)	6.6 (47, 666)	2.9 (4, 135)	6.3 (60, 890)	3.7 (33, 870)	1E-68	1E-52	P	319	19.9
*Peg10*	chr6	0.1 (18, 12098)	0.2 (226, 149701)	0.3 (310, 123681)	0.1 (4, 3763)	0.3 (507, 150895)	6.0 (4951, 78040)	0E+00	4E-10	P	29	793
*Mest*	chr6	0.0 (0, 16)	1.7 (3, 175)	0.3 (11, 3209)	0.4 (2, 447)	0.5 (13, 2780)	1.1 (71, 6602)	3E-117	4E-105	P	42	65.1
*Peg3*	chr7	0.2 (7, 4413)	0.1 (21, 24916)	0.2 (44, 19674)	0.5 (17, 3529)	19.6 (4032, 16588)	41.4 (10215, 14486)	1E-302	2E-02	P	107	75.2
*Usp29*	chr7	4.1 (4, 94)	1.7 (14, 829)	2.2 (8, 361)	2.2 (1, 44)	16.9 (76, 374)	42.3 (80, 109)	2E-87	4E-04	P	237	2.56
*D7ertd715e**^a^*	chr7	0.0 (0, 86)	0.0 (0, 313)	0.0 (0, 243)	0.0 (0, 95)	1.1 (2, 176)	0.0 (0, 78)	4E-69	3E-37	P	64	5.96
*Snrpn*	chr7	0.0 (0, 48)	0.5 (1, 208)	1.4 (2, 146)	9.1 (1, 10)	2.0 (2, 97)	7.7 (3, 36)	3E-45	2E-14	P	97	13.6
*H19**^a^*	chr7	95.5 (2508, 119)	95.4 (8222, 394)	95.7 (15727, 714)	98.8 (61749, 756)	99.6 (170049, 742)	51.3 (173851, 164950)	7E-242	2E-03	M	9	2432
*Ascl2*	chr7	92.6 (50, 4)	89.9 (519, 58)	86.1 (352, 57)	100.0 (10, 0)	81.6 (476, 107)	96.2 (101, 4)	1E-40	6E-08	M	4	13.6
*Cd81*	chr7	67.0 (254, 125)	90.3 (1678, 181)	80.9 (2438, 576)	72.3 (225, 86)	79.5 (2277, 587)	81.5 (2043, 465)	8E-11	3E-17	M	74	87.8
*Tssc4*	chr7	65.3 (47, 25)	82.7 (382, 80)	79.1 (453, 120)	60.9 (53, 34)	75.6 (436, 141)	69.6 (385, 168)	1E-14	2E-04	M	9	3.30
*Kcnq1ot1**^a^*	chr7	0.2 (1, 544)	0.2 (2, 1139)	5.3 (56, 1010)	0.3 (1, 349)	0.5 (3, 580)	0.0 (0, 305)	9E-18	8E-87	P	371	0.99
*Cdkn1c*	chr7	99.7 (1185, 3)	100.0 (9367, 4)	99.8 (6716, 13)	100.0 (351, 0)	76.5 (6925, 2123)	99.9 (2673, 4)	0E+00	9E-06	M	3	335
*Slc22a18*	chr7	88.5 (46, 6)	89.8 (264, 30)	88.7 (235, 30)	92.9 (13, 1)	72.6 (244, 92)	98.7 (155, 2)	6E-33	2E-04	M	141	6.75
*Phlda2*	chr7	100.0 (120, 0)	100.0 (4921, 1)	99.3 (3279, 24)	95.7 (112, 5)	72.2 (3611, 1392)	98.7 (1211, 16)	2E-55	1E-06	M	2	290
*Gab1*	chr8	15.3 (342, 1896)	18.3 (1745, 7809)	14.8 (1624, 9329)	9.2 (129, 1275)	24.8 (2367, 7163)	16.8 (1575, 7804)	3.E-66	2.E-12	P	633	58.3
*Plagl1*	chr10	0.0 (0, 86)	0.4 (2, 528)	1.0 (2, 202)	0.0 (0, 19)	1.3 (4, 297)	4.2 (4, 91)	4E-76	1E-27	P	142	1.15
*Grb10*	chr11	68.8 (1527, 691)	89.1 (5640, 692)	85.0 (5615, 994)	98.2 (1849, 33)	92.0 (8908, 777)	86.4 (6314, 994)	5E-14	7E-12	M	455	159
*Zrsr1*	chr11	0.0 (0, 131)	0.4 (1, 225)	0.4 (1, 273)	0.0 (0, 33)	21.4 (69, 254)	1.9 (6, 312)	4E-72	4E-08	P	7	4.86
*Grb10as**^a^*	chr11	69.9 (51, 22)	87.9 (94, 13)	92.9 (79, 6)	97.3 (36, 1)	88.7 (94, 12)	87.2 (75, 11)	4E-08	1E-12	M	123	0.08
*Dlk1*	chr12	100.0 (2, 0)	99.5 (195, 1)	93.1 (297, 22)	100.0 (2, 0)	93.8 (45, 3)	98.1 (53, 1)	2E-18	2E-10	P	15	1.47
*Meg3**^a^*	chr12	99.3 (438, 3)	70.0 (7, 3)	97.6 (290, 7)	99.9 (1392, 1)	99.8 (3325, 5)	99.9 (11062, 8)	2E-69	6E-126	M	109	85.3
*Mirg**^a^*	chr12	100.0 (159, 0)	100.0 (10, 0)	93.1 (54, 4)	100.0 (772, 0)	100.0 (716, 0)	99.8 (2007, 4)	1E-24	4E-150	M	119	11.4
*Pde10a*	chr17	90.9 (251, 25)	88.3 (878, 116)	85.6 (600, 101)	97.1 (34, 1)	73.8 (479, 170)	77.0 (349, 104)	5E-64	1E-06	M	448	3.10
*Slc22a3*	chr17	100.0 (41, 0)	98.2 (667, 12)	93.6 (612, 42)	23.1 (3, 10)	96.7 (353, 12)	96.8 (91, 3)	9E-40	1E-02	M	143	2.05
*Airn**^a^*	chr17	4.3 (28, 628)	0.4 (19, 5256)	0.4 (18, 4213)	1.6 (6, 370)	1.4 (47, 3304)	0.8 (11, 1362)	2E-30	9E-124	P	443	3.01
*Igf2r*	chr17	97.9 (1011, 22)	98.6 (2650, 38)	98.4 (3821, 63)	97.8 (723, 16)	98.6 (3348, 48)	98.8 (4743, 57)	1E-199	2E-111	M	330	73.0
*Igf2*	chr7	45.8 (2694, 3186)	32.5 (22266, 46340)	29.0 (20372, 49913)	8.2 (71, 790)	46.9 (17649, 19980)	75.9 (24740, 7869)	1E-4	6E-1	NS	21	1418

Gene ID and chromosomal location are listed. For each CB.x and BC.x cell line, the percent of allele-specific reads mapping to the maternally inherited chromosome is shown. In that same column, the absolute number of maternal and paternal SNP-overlapping read counts detected in each TSC line, respectively, are shown in parentheses. *P*-values for corresponding PO biases in CB.x and BC.x cell lines were corrected for multiple testing via Benjamini Hochberg, and are listed under “CBp” and “BCp” columns, respectively. B, parental direction of PO bias: P, paternally biased; M, maternally biased; NS, not significantly biased. PO, parent-of-origin; TSC, trophoblast stem cell; SNPs, single-nucleotide polymorphisms—the number of distinct SNPs covered by RNA-Seq reads for the respective transcript. RPKM, average allele-nonspecific RPKM (reads per kilobase of transcript per million aligned reads) of each respective transcript across the 6 profiled TSC lines; lncRNA, long noncoding RNA.

a Known or putative lncRNAs.

**Table 3:** Candidate imprinted genes expressed with significant PO bias in TSCs

Gene	Chr	Coords	S	CB.1	CB.2	CB.3	BC.1	BC.2	BC.3	CBp	BCp	B	SNPs	RPKM
*Id1*	chr2	152562009-152563146	+	43.1 (31, 41)	33.5 (52, 103)	35.3 (55, 101)	35.2 (32, 59)	35.5 (39, 71)	15.4 (14, 77)	2.E-02	9.E-04	P	4	16.0
R74862*	chr7	150207688-150239273	−	65.8 (25, 13)	87.7 (71, 10)	82.5 (99, 21)	58.3 (21, 15)	72.4 (63, 24)	72.0 (67, 26)	1.E-07	1.E-02	M	98	0.27
Tsci1*	chr8	83383307-83405773	+	0.0 (0, 41)	0.7 (1, 148)	0.7 (1, 143)	0.0 (0, 40)	12.5 (10, 70)	9.5 (13, 124)	3.E-40	3.E-15	P	82	0.16
Tsci2*	chr11	12051323-12097365	+	57.6 (19, 14)	85.7 (48, 8)	77.1 (37, 11)	95.5 (21, 1)	93.5 (87, 6)	97.9 (93, 2)	8.E-04	1.E-16	M	75	0.08
*AK017220*	chr11	51003334-51033915	−	3.6 (6, 159)	2.2 (3, 134)	0.7 (1, 137)	4.8 (4, 79)	56.3 (117, 91)	7.2 (15, 192)	3.E-44	4.E-03	P	161	2.10
*Tsci3*	chr11	51033968-51061895	+	3.9 (5, 123)	3.1 (9, 280)	0.3 (1, 285)	4.4 (2, 43)	46.9 (207, 234)	9.6 (42, 396)	7.E-54	4.E-04	P	101	0.32
AK010368*	chr11	97459824-97484207	+	0.7 (1, 133)	1.5 (6, 407)	0.7 (3, 451)	1.5 (1, 64)	37.0 (101, 172)	47.4 (451, 500)	5.E-92	3.E-02	P	150	4.40
Tsci4*	chr12	82108796-82120245	−	5.6 (1, 17)	0.0 (0, 40)	0.0 (0, 45)	0.0 (0, 10)	15.4 (2, 11)	48.3 (14, 15)	3.E-17	5.E-02	P	52	0.04
Tsci5*	chr12	110730857-110753078	−	100.0 (2, 0)	100.0 (8, 0)	100.0 (16, 0)	100.0 (6, 0)	100.0 (12, 0)	100.0 (34, 0)	2.E-07	1.E-10	M	35	0.03
*Pdgfb*	chr15	79826305-79845238	−	41.3 (250, 356)	42.2 (1999, 2737)	38.6 (2126, 3380)	27.9 (58, 150)	48.8 (2928, 3070)	44.2 (3471, 4375)	6.E-03	4.E-02	P	97	62.3
Tsci6*	chr15	96980667-96997795	−	7.8 (17, 200)	19.7 (12, 49)	3.5 (12, 333)	0.3 (1, 386)	0.9 (6, 662)	0.9 (9, 1032)	8.E-18	5.E-103	P	129	0.75
*1700010i14rik*	chr17	9181197-9201184	+	100.0 (18, 0)	84.0 (21, 4)	90.7 (39, 4)	100.0 (9, 0)	100.0 (33, 0)	70.1 (47, 20)	8.E-11	2.E-03	M	43	0.81
*Qk*	chr17	10399335-10512226	−	55.2 (235, 191)	56.4 (648, 501)	55.2 (574, 465)	70.2 (342, 145)	64.3 (687, 381)	63.9 (565, 319)	7.E-03	1.E-04	M	176	36.2
*Pacrg*	chr17	10595877-11033057	−	75.0 (3, 1)	92.9 (13, 1)	86.7 (13, 2)	100.0 (5, 0)	90.0 (9, 1)	84.6 (11, 2)	1.E-04	9.E-04	M	29	0.21
*Park2*	chr17	11033249-12256226	+	86.8 (105, 16)	81.3 (74, 17)	85.7 (60, 10)	61.1 (33, 21)	76.5 (78, 24)	73.6 (53, 19)	9.E-18	3.E-03	M	273	0.08
*Mas1*	chr17	13033870-13061009	−	100.0 (6, 0)	100.0 (63, 0)	99.2 (123, 1)	100.0 (2, 0)	100.0 (34, 0)	100.0 (12, 0)	2.E-27	4.E-10	M	55	0.27
*Dact2*	chr17	14332236-14340838	−	59.6 (56, 38)	56.9 (357, 270)	54.5 (486, 405)	83.2 (84, 17)	71.4 (401, 161)	70.8 (685, 283)	3.E-03	6.E-07	M	40	7.65

Column annotations are the same as in Table 2. Coordinates given are relative to UCSC’s mm9 genome build. PO, parent-of-origin; TSC, trophoblast stem cell; SNP, single-nucleotide polymorphism; RPKM, reads per kilobase of transcript per million aligned reads.

Notable known imprinted genes with significant PO expression biases in TSCs included the *Kcnq1ot1* imprinted lncRNA and many of its nearby target genes (*Cdkn1c*, *Cd81*, *Phlda2*, *Slc22a18*, *Tssc4*), the *Airn* imprinted lncRNA and two of its nearby target genes (*Igf2r* and *Slc22a3*), *H19*, *Grb10*, *Meg3*, *Mirg*, *Gab1* (Okae *et al.* 2012), and *Sfmbt2* and its antisense noncoding transcript, *AK076687* (Wang *et al.* 2011a). Another PO-biased gene, annotated as *D7ertd715e* in the mouse, is syntenic to a complex series of lncRNA transcripts that originate from the imprinted Prader-Willi locus on human chromosome 15, and may be a mouse homolog, or it may be an 3′ extension of the neighboring imprinted gene, *Snrpn*. The *D7ertd715e* transcript was recently reported to be imprinted in trophoblast cells derived from horse/donkey F1-hybrids (Wang *et al.* 2013b).

An additional 41 known imprinted genes present in MRC Harwell’s Imprinting Resource were expressed in TSCs with enough allelic coverage to pass our threshold for analysis but were not detected as significantly PO biased (Williamson *et al.* 2013) (Table S1). Many of these genes, such as *Wt1*, *Ube3a*, *Rasgrf1*, and *Zdbf2*, were neutrally biallelic across the profiled TSC lines, and at least four, *Pon2*, *Klf14*, *Atp10a*, and *Art5*, were expressed with significant strain-of-origin bias (as opposed to a PO bias), underscoring the tissue-specificity with which imprinted gene expression is known to occur (Prickett and Oakey 2012).

**Figure 2:**
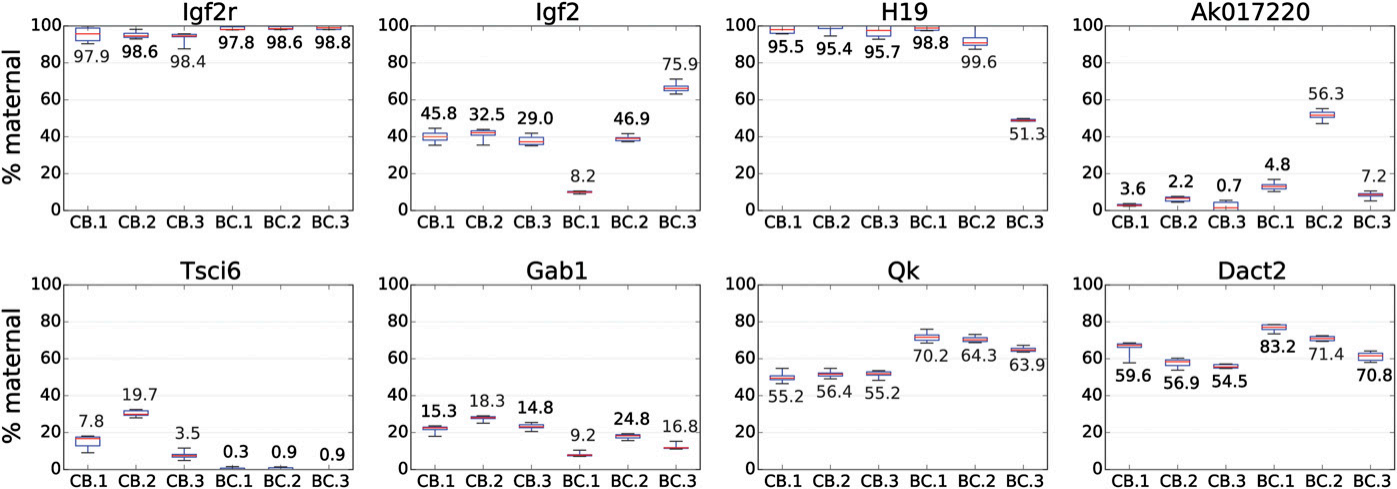
Detection of allele-specific expression by pyrosequencing. Individual subplots show maternal expression in six reciprocally derived F1-hybrid TSC lines for eight genes assayed. CB.1, CB.2, and CB.3 were derived from a Cast mother and B6 father, whereas BC.1, BC.2, and BC.3 were derived from a B6 mother and Cast father. Box and whisker plots represent data collected from six technical replicates, except in the H19 subplot, where data derived from four technical replicates. For comparison, numerical values above/below each box and whisker plot show the corresponding maternal expression percentages determined from RNA-Seq in each TSC line. TSC, trophoblast stem cell.

To gain a sense of the accuracy with which our RNA-Seq analysis pipeline detected PO biases in TSCs, we re-measured PO expression bias for eight genes using QIAGEN’s PyroMark pyrosequencing assay. These eight genes included three known imprinted genes expressed with significant PO bias in TSCs (*Igf2r*, *H19*, and *Gab1*), one known imprinted gene whose PO expression bias in TSCs was not called as significant in our analysis (*Igf2*), and four PO biased genes that to our knowledge have not been previously reported as imprinted. Pyrosequencing primers were designed around single informative SNPs contained within each gene and assays were performed per gene in each of the six TSC lines profiled for RNA-Seq. In all 48 cases (eight genes in six TSC lines), allelic biases determined via RNA-Seq and pyroseqeuncing were concordant (Figure 2). This high level of concordance mirrors that observed in our previous analysis of X-linked gene expression in TSCs, where allelic biases determined via RNA-Seq and an alternate method were concordant in 18 of 18 assays (nine genes in two TSC lines) (Calabrese *et al.* 2012). Considering the data shown in Figure 2 and in our previous work, we conclude that the majority of allelic measurements reported by our RNA-Seq analysis are accurate approximations of steady state gene expression levels present in TSCs.

### General characteristics of known and candidate imprinted TSC genes

Many of the 48 PO biased genes displayed variability in their PO expression bias across our panel of TSC lines, including *Sfmbt2*, *Phf17*, *Peg3*, *Usp29*, *Cd81*, and *Peg2* (Table 2). However, this variability was most notable for *H19* and *Igf2*, two neighboring genes whose imprinting status is conserved between human and mouse. *H19* is maternally expressed, and *Igf2* paternally expressed, in both species (Fedoriw *et al.* 2012a). *H19* was expressed at >95% from the maternal allele in all but one profiled TSC line, BC.3, where its maternal-to-paternal expression ratio was 51-to-49 (Table 2). The variation in PO expression bias of the neighboring paternally biased *Igf2* was even more pronounced than that of *H19*, to the extent that significant PO expression bias was not detected for *Igf2* in our panel of TSCs. Paternal expression of *Igf2* ranged from a low of 24% in BC.3 (meaning it was *maternally* biased in that TSC line), to a high of 92% in BC.1 (Table 2). Notably, the TSC line that displayed the near equal maternal-to-paternal expression ratio for *H19*, TSC line BC.3, was the same line that displayed a maternal expression bias for *Igf2* (Table 2).

Although no other known imprinted gene displayed a variation in PO bias as dramatic as *Igf2*, a handful of known imprinted genes, such as *Osbpl5*, *Impact*, *Dhcr7*, and *Ddc*, also were expressed with a mild PO bias in only five of six profiled TSC lines and as a result were not detected as significantly biased by edgeR (Table S1). Interindividual variation in imprinted gene expression was observed recently in mule and hinny trophoblast cells (Wang *et al.* 2013b), and its documentation here in mouse TSCs lends support to the idea that such variation may be a conserved feature of genomic imprinting across mammals. Although it remains to be tested in future studies, we speculate that at least some of the variability in imprinted gene expression that we observed in TSCs is due to stochastic variation in levels of DNA methylation or other epigenetic marks at imprinted control regions; this putative variation could have been acquired in cell culture, or may have been naturally present in individual trophoblast cells at the time of TSC derivation. In either case, the observed variation in imprinted gene expression supports the recently proposed notion that genomic imprinting may have evolved as a means to confer robustness to developing embryos during changes in fetal environmental conditions (Radford *et al.* 2011). Further, the presence of such variation supports our strategy to detect consistently imprinted transcripts by profiling PO biased expression across multiple TSC lines.

We detected significant PO expression biases for 17 genes not previously reported to be subject to genomic imprinting, referred to hereafter as candidate imprinted genes (CIGs; Table 3). In general, total expression levels of the CIGs were lower than those of the known imprinted genes. Average and median expression levels for the CIGs were 7.7 and 0.3 RPKM, respectively, compared with 190 and 13.6 RPKM for known imprinted genes (Tables 2 and Table 3). Like that observed for the known imprinted genes, some CIGs, such as *Tsci1* and *Tsci6*, showed strong PO bias in all six TSC lines profiled, whereas others exhibited more mild levels of PO bias (Table 3). For example, *Qk* had an average maternal bias in the CB.x lines of 55%, and an average maternal bias in the BC.x lines of 66%, and all of these values were confirmed via pyrosequencing (Figure 2). We speculate that certain genes with mild PO expression biases in TSCs, such as the CIG *Qk*, may not be imprinted in the canonical sense, but may exhibit imprinted expression due to their proximity to strongly imprinted controlling elements that are able to impose a PO expression bias on nearby susceptible genes.

**Figure 3:**
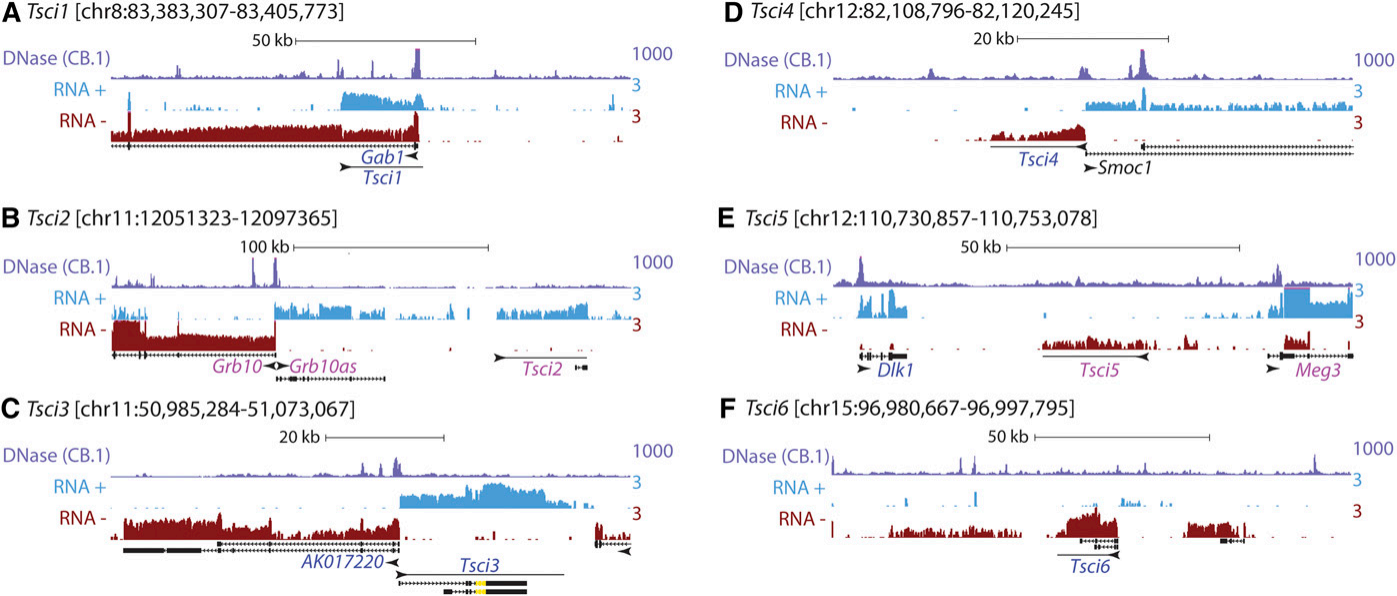
Genomic environment surrounding *Tsci* CIG transcripts. (A−F) Name and genomic coordinates for each *Tsci* transcript relative to UCSC Genome Build mm9. Shown are wiggle density profiles of TSC RNA-Seq data pooled from all six profiled lines, partitioned by matching genomic strand (RNA “+” or “−”), as well as CB.1 DNaseI Hypersensitivity density from (Calabrese *et al.* 2012). The names of the University of California Santa Cruz Known Genes and *Tsci* transcripts are indicated above or below their genomic locations. Transcript names in pink and blue signify genes expressed with significant maternal and paternal biases in TSCs, respectively. Arrowheads indicate direction of transcription detected via RNA-Seq. Box-and-wishbone structures indicate splice-forms annotated in the UCSC database or those detected via Cufflinks analysis of pooled TSC RNA-Seq data. RNA-Seq read count density has been log-10 transformed, DNaseI I read count density has not. In (C), the yellow portion of the box-and-wishbone structure of *Tsci3* corresponds to the location of the Ensembl noncoding Gene, *ENSMUSG00000061469*. CIG, candidate imprinted genes. TSC, trophoblast stem cell.

We detected six CIGs that are not currently annotated as transcribed regions or genes in the mm9 or mm10 UCSC builds of the mouse genome (Kent *et al.* 2002). We have provisionally named these transcripts TSC Imprinted (*i.e.*, *Tsci*) 1 through 6 (Table 3 and Figure 3). A minority fraction of one of these transcripts, *Tsci3*, overlapped with a nonprotein coding gene annotated in the Ensembl genome database, *ENSMUSG00000061469* (Cunningham *et al.* 2014) (yellow box-and-wishbone in Figure 3C). The remaining five *Tsci* transcripts had no corresponding annotations in UCSC, Ensembl, or GENCODE builds of the mouse transcriptome (Kent *et al.* 2002; Harrow *et al.* 2012; Cunningham *et al.* 2014).

We assessed the coding potential of the six *Tsci* transcripts using two prediction algorithms, CPAT and CPC, and found that the *Tsci3* transcript has potential to encode for a 225 amino acid hypothetical protein that does not appear to be conserved in human (Kong *et al.* 2007; Wang *et al.* 2013a). Notably, *Tsci3* was transcribed divergently from another hypothetical protein, *AK017220*, which itself was detected as a CIG in TSCs (Table 3). We suggest the remaining five *Tsci* transcripts are putative lncRNAs given their length of greater than 200 nucleotides and their lack of coding potential. One of these transcripts, *Tsci2*, has a transcribed counterpart in its syntenic human region, the GENCODE lncRNA RP4-724E13.2 (Harrow *et al.* 2012); the other five do not.

Our identification the *Tsci* transcripts supports efforts put forth by the ENCODE consortium and others to perform RNA-Seq across large panels of cell and tissues (Yue *et al.* 2014). LncRNAs are known to be expressed with a tissue specificity equal to that of protein coding genes (Cabili *et al.* 2011; Derrien *et al.* 2012), but at present their locations in genomes cannot be predicted, and thus their existence must be determined via empirical measurement. On the basis of our discovery of the *Tsci* transcripts in this work, it seems likely that continued RNA-Seq profiling in rare or understudied cell populations might also uncover new lncRNAs. By this same logic, similar profiling efforts performed in F1-hybrid backgrounds may uncover additional tissue-specific imprinted transcripts.

Consistent with the tendency of imprinted genes to be localized within clusters in the genome, 13 of 17 CIGs were located near known imprinted genes or other CIGs (Figure 3 and not shown). These include the *R74862* lncRNA, located within the cluster of maternally biased genes surrounding the *Kcnq1ot1* lncRNA, *Gab1* and *Tsci1*, which are divergent transcript pairs, *Tsci2*, located adjacent to *Grb10* and *Grb10as*, *AK017220* and *Tsci3*, also divergent transcript pairs, *Tsci4*, located between the known imprinted genes *Dlk1* and *Meg3*, and six CIGs surrounding the known imprinted genes *Pde10a* and *Airn* (*1700010I14Rik*, *Qk*, *Pacrg*, *Park2*, *Mas1*, and *Dact2*).

We next examined the 24 maternally and paternally biased genes, respectively, for significant enrichment in functional ontologies using the DAVID tool (Huang da *et al.* 2009), and found none after correction for multiple testing. However, visual inspection of the list of PO biased genes yielded at least two notable insights. Foremost, six of the 17 CIGs are associated with human disease. These include *Id1* (prostate cancer), *Pdgfb* (dermatofibrosarcoma protuberans, meningioma), *Qk* (schizophrenia, 6q terminal deletion syndrome), *Pacrg* (Parkinson’s disease, leprosy), *Park2* (Parkinson’s disease, cancer), and *Mas1* (hypotension, cancer) (Rebhan *et al.* 1998; OMIM 2013). Collectively, these six genes either had weak PO biases or were lowly expressed and it is not immediately clear what biological role if any their candidate imprinting plays in TSCs (Table 3). Nevertheless, it remains possible that in certain scenarios or cell types their PO expression bias may fluctuate to fulfill a physiologically important function.

Second, we found that 19 known and CIGs expressed in TSCs are nonprotein coding and/or have potential to express microRNAs, supporting the notion that noncoding RNAs play integral roles in aspects of TSC biology. In total, 16 of 48 genes expressed with significant PO bias in TSCs appeared to be lncRNAs. Of these 16 lncRNAs, only the *Kcnq1ot1* and *Airn* lncRNAs were surrounded by genes with opposing imprints (*i.e.*, a paternally biased lncRNA surrounded by maternally biased genes), suggesting the remaining 14 lncRNAs have biological functions other than localized, allele-specific transcriptional repression. Three known imprinted lncRNA transcripts expressed in TSCs are embedded with microRNAs (*H19*, *Mirg*, and *Meg3*), as are the introns of an additional three imprinted, expressed protein coding genes (*Sfmbt2*, *Mest*, *Usp29*). In total, these six transcripts encode for 60 known microRNAs (Table 4).

**Table 4:** Imprinted genes expressed in TSCs that encode miRNAs

Host Gene	miRNAs
*Sfmbt2*	*miR669a-3*, *miR467d*, *miR669a-2.5*, *miR297b*, *miR669a-2.2*, *miR669e*, *miR669a-2.9*, *miR467a-1.9*, *miR466*, *miR466g*, *miR467a-1.5*, *miR4660*, *miR669a-2.8*, *miR467a-1.10*, *miR297a-3*, *miR669a-2.7*, *miR467a-1.1*, *miR669h*, *miR669a-2.1*, *miR669g*, *miR669a-1*, *miR699p-1.1*, *miR669c*, *miR467a-1.6*, *miR669m-1*, *miR669a-2.4*, *miR669a-2.10*, *miR699p-1.2*, *miR467a-1.2*, *miR669k*, *miR467a-1.8*, *miR669a-2.6*, *miR467a-3*, *miR669a-2.3*, *miR669i*, *miR467a-1.7*, *miR669b*, *miR467b*, *miR669j*, *miR467a-1.3*, *miR467a-1.4*
*Mest*	*miR335*
*Usp29*	*miR3099*
*H19*	*miR675*
*Meg3*	*miR1906-1*, *miR770*
*Mirg*	*miR377*, *miR134*, *miR496*, *miR154*, *miR412*, *miR485*, *miR382*, *miR410*, *miR668*, *miR3072*, *miR453*, *miR409*, *miR541*, *miR369*

TSCs, trophoblast stem cells, miRNA, microRNA.

Proper imprinted gene expression is essential for mammalian development and its misregulation plays major roles in several human diseases, including many types of cancers and Beckwith-Wiedemann, Silver-Russell, Angelman, and Prader-Willi syndromes (Butler 2009; Barlow 2011; Ferguson-Smith 2011; Garfield *et al.* 2011; Lee and Bartolomei 2013). The focused study of the mechanisms by which imprinted gene expression is established and maintained may therefore yield important insights into human development and the molecular etiology of these diseases, and, more broadly, may shed light on important principles that govern the epigenetic regulation of gene expression.

We report the first genome-wide assessment of imprinted gene expression in mouse TSCs. Our data indicate that TSCs are robustly subject to genomic imprinting, including in regions known to be silenced by the imprinted lncRNAs *Kcnq1ot* and *Airn*, similar to that observed in prior studies examining TSC imprinted expression via single gene assays (Lewis *et al.* 2006; Fedoriw *et al.* 2012b; Miri *et al.* 2013). TSCs also expressed high levels of several imprinted lncRNAs and transcripts that are known microRNA precursors, including *H19*, *Mirg*, and *Meg3*, suggesting an integral role for noncoding RNA in TSC biology. Our allele-specific expression maps and the F1-hybrid TSCs from which they were derived represent a resource to dissect the mechanisms that cause imprinted gene expression in the mouse, and the cell autonomous roles that imprinted genes play in TSC biology.
